# Swedenborg and Medicine

**Published:** 1892-03

**Authors:** Chas. S. Mack

**Affiliations:** Ann Arbor, Michigan


### THE VITAL FORCE DEFINED.

### SWEDENBORG AND MEDICINE.

CHAS. S. MACK, M. D., ANN ARBOR, MICHIGAN.

It is but a few minutes since the postman handed me THE HOMŒOPATHIC PHYSICIAN, for February, and in it I have read with unusual interest the late Dr. Bayard's remarks, under heading "The Vital Force Defined." The occasion of my writing is that Dr. Bayard quotes Swedenborg. For some few years I have been a reader of Swedenborg, and have believed that in his writings are expressed the truths of a new age. It has seemed to me that Homœopathy (than which nothing could be more radically revolutionary) is in harmony with this new age. In a little book entitled *Philosophy in Homœopathy* (published by Gross & Delbridge, 48 Madison St., Chicago), I have attempted to bring the light of this new age to bear upon *Similia similibus curantur;* and I suspect that through the writings of Swedenborg we may get light upon various subjects—*e. g.*, the subject of dosage and of the dynamic power of drugs.

Emerson rejected the view that Swedenborg was the commissioned revelator of doctrines to a new age, but in his lecture *Swedenborg, or the Mystic* in his *Representative Men,* he credits Swedenborg with genius and learning most extraordinary. Among other things he says that Swedenborg "must be reckoned a leader in that revolution, which, by giving to scienc, an idea, has given to an aimless accumulation of experiments: guidance and form, and a beating heart."

I believe that these are the best of reasons for the fact often observed, that New Church people are apt to believe in Homœopathy. To any of your readers who is wondering whether Swedenborg has anything to say of practical value to the medical world, I cannot too cordially or too enthusiastically say, *read him.*

